# Explanation and verification of the rules of attack in table tennis tactics

**DOI:** 10.1186/s13102-022-00396-3

**Published:** 2022-01-08

**Authors:** Xingdong Zhou

**Affiliations:** Faculty of Sports Science, Ningbo University, No. 818 Fenghua Road, Ningbo, 315211 Zhejiang China

**Keywords:** Table tennis, The skills and tactics, Rules of attack

## Abstract

**Background:**

The rules of attack in table tennis tactics have been discovered by the coaches and researchers of the Chinese table tennis team (CTTT) through long-term practice. However, they are only empirical judgements and have not been objectively verified.

**Methods:**

The software "Table Tennis Master" has been used to analyse 200 matches of top players of CTTT against various opponents in recent years to obtain detailed statistics by analysing the effect of attack in the end line (AIEL) and attack out of the end line (AOEL).

**Results:**

(1) The scoring rate of the players was high after AIEL but very low after AOEL (*p *< 0.05); (2) the round of service (serve/receive) and level of skills had little influence on the effect of AIEL and AOEL; and (3) the timing of attack had a great influence on the effect of AIEL and AOEL (r > 0.9).

**Conclusions:**

In the high-level table tennis match, the rules of AIEL and AOEL are scientific. In accordance with the rules, the complex tactics can be simplified to the two concepts, AIEL and AOEL.

## Background

For nearly half a century, China has always occupied the dominant position in the world table tennis competition. Owing to skilled athletes and experienced coaches, as well as their exploration of advanced skills, the Chinese table tennis team (CTTT) has accomplished great achievements in international competition. To continue to rank first in the world, CTTT has done a lot, including researching skills and innovating.

Table tennis is a skills and tactics oriented event, and the key factors in high-level competition should always be skills and tactics [1–6]. Their status should be higher than that of physical ability [7], psychological ability [8] and other factors. However, there are various types of skills and tactics in table tennis [9, 10]. Although there are many skills and tactics analysis methods [1], they are still very complex. Therefore, it is difficult for athletes and coaches to master better skills and tactics before matches.

After long-term research on the CTTT and in-depth discussion with its coaches and athletes, the rules of attack in table tennis tactics are formed. In the high-level table tennis matches, the effect of attack in the end line (AIEL) is very good, while the effect of attack out of the end line (AOEL) is bad. If the tactical strategies are well arranged in advance according to the rules of attack position, it can obtain better results. However, this fact is only an empirical judgement of coaches and researchers, and there is no objective method to verify the rules and explore the different effects between AIEL and AOEL. This paper researches the skills and tactical performance of top players of CTTT in matches (6 male athletes, right-handed, attacker, the average age is 25, ranked top 30 in the world) and aims to analyse their 200 matches against varying levels of opponents in international matches in recent years, including 100 matches against high-level opponents (ranked top 30 in the world by the International Table Tennis Federation, HO) and 100 matches against low-level opponents (ranked below 30 in the world by the International Table Tennis Federation, LO).

The author hopes that this study will be helpful in the formulation of skills and tactics for top-level table tennis players and their training in the future. At the same time, it will be beneficial to transform the rules founded by CTTT in practice to the theoretical level and to make contributions to the development of table tennis skills and tactical theory.

## Methods

### Video observation method

Videos of the top players of the CTTT against opponents of varying world rankings in international matches are viewed to intuitively understand the characteristics of their skills and tactics in the matches, especially the effects of the AIEL and AOEL. A total of 200 recent match videos were selected, including 100 matches against HO and 100 matches against LO. The method is the same as that of Huang [11] and Guo [12]. In this process, the intuitive feelings of six Chinese table tennis team coaches were collected after they watched the videos, which provides a reference for the analysis of the results.

### Statistical method of table tennis skills and tactics

#### Introduction to the statistical method of table tennis skills and tactics

The software "Table Tennis Master" was used to record, count and classify the skills and tactics data of the players in the matches [3]. All data were collected by the author, which ensured the consistency of the data collection standards. After the data collection, two professional table tennis match data analysts verified the data, which ensured reliability.

To analyse the characteristics of top players' skills and tactics in the matches, the method is the same as that of Huang [11] which makes statistical analysis of the effect of AIEL and AOEL more detailed. This analysis is performed using the serve-then-attack section (including the service, the third stroke, the fifth stroke, STAS), the receive-then-attack section (including the receive and the fourth stroke, RTAS), and the stalemate section (the sixth stroke and later, SS).

In the statistical method of table tennis skills and tactics, two indices of scoring rate and usage rate were selected [13, 14]. The calculation methods of these two indices are as follows.

The scoring rate of a certain evaluation index = scores won from this index/ (scores won from this index + scores lost from this index).

The usage rate of a certain evaluation index = (scores won from this index + scores lost from this index)/ (all scores won + all scores lost).

#### The definition of some concepts when using the statistical method

According to the author's understanding, some concepts of table tennis skills and tactics have not yet been unified around the world. To ensure that the readers can achieve a unified understanding, it is necessary to define some concepts used in this study.

**Fig. 1:**
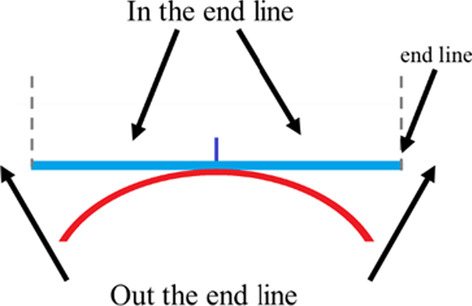
The attack position

**Fig. 2:**
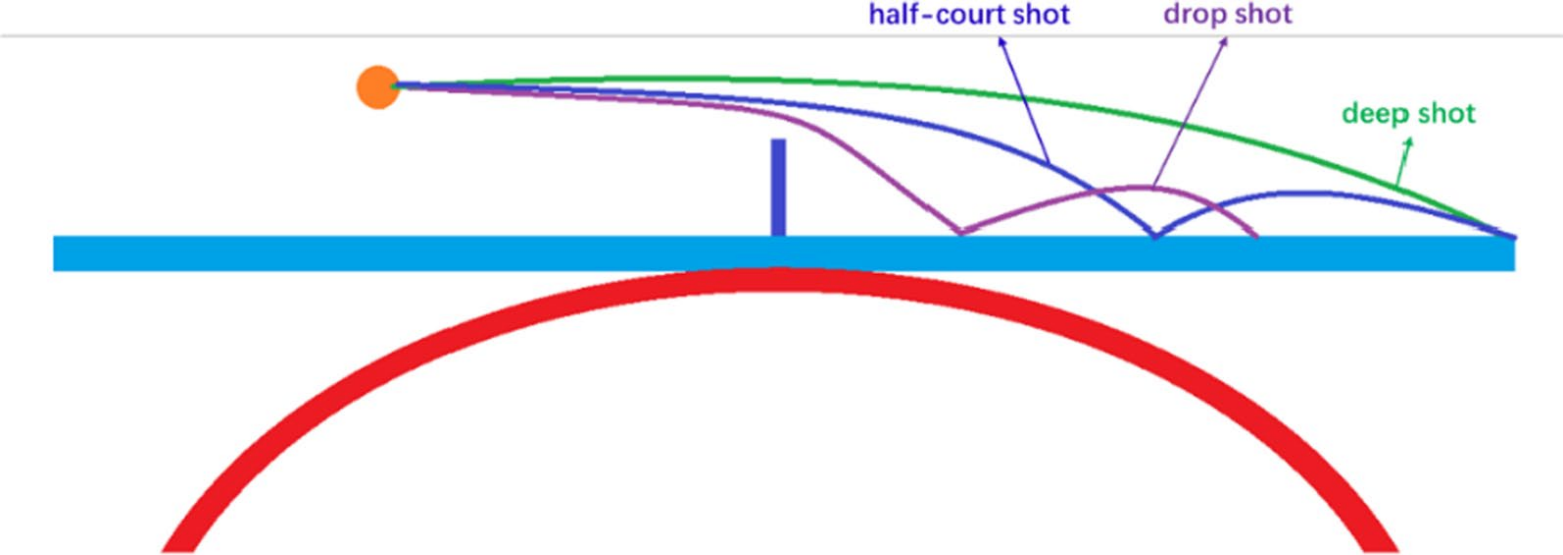
Schematic diagram of ball movement after hitting with different skills

1. Attack: A collective of table tennis skills and tactics that converts the ball from control confrontation to topspin confrontation in each rally of a match. In this study, the "attack" can be divided into attacks in the end line and attacks out of the end line, according to different positions.
2. Attack in the end line (AIEL): Attack when the ball lands near the net or half-court, and the position of the racket directs the ball in the end line (Fig. 1). For example, attack when the opponent’s service is short, service half-court shot, drop shot, drop half-court shot, and so on.
3. Attack out of the end line (AOEL): Attack when the ball lands near the end line, and the position of the racket directs the ball out of the end line (Fig. 1). For example, attack when the opponent’s service is long, deep shot, and so on.
4. Drop shot: Push the ball in the end line, and the ball lands near the net with the second landing point on the table (Fig. 2).
5. Deep shot: Push the ball in the end line, and the ball lands near the end line (Fig. 2).
6. Half-court shot: Serve or push the ball, and the ball lands near the half-court with the second landing point just near the end line (Fig. 2).

### Statistical analysis

All data tests were performed by using SPSS version 21.0 software (SPSS Inc., Chicago, IL, USA) for Windows. One-way analysis of variance was used to examine the differences in the scoring rate after attacking between different attack positions, different skills, and different groups. The Pearson correlation coefficient was used to examine the relationship between the attack timing and the scoring rate. The significance level for all tests was set at p<0.05.

## Results

### The scoring rate after attacking at different positions

The scoring rate after attacking is based on different positions. When players of CTTT play against HO, the scoring rate after AIEL is 66.0%. When they play against LO, the scoring rate after AIEL is 72.9%. However, the scoring rate will be 13.2% and 14.9% respectively after AOEL. It is obvious that the scoring rate of AIEL is much higher than that of AOEL, whether they play against the HO or LO.

On the other hand, the scoring rate of CTTT players was 31.8% after AIEL according to the HO. In addition, it is 71.6%, after AOEL by the same opponents. Similarly, it is the same rule when the LO attack. This shows that there is a large difference in the scoring rate among different attack positions, and there is no statistical significance regarding the level of the opponents.

### The relationship between the rounds and scoring rate after attacking at different positions

Regardless of whether they are in the serve rounds or in the receive rounds, players of CTTT have a high AIEL scoring rate when they play against opponents in different level groups. The scoring rate is more than 70% in the serve rounds, and it is more than 60% in the receive rounds. Meanwhile, the difference in scoring rate in the serve rounds is larger than that of the receive rounds among the opponents in different level groups. In terms of AOEL, they have a low scoring rate whether they are in the serve rounds or in the receive rounds.

In general, the rounds do not affect the high scoring rate after AIEL, nor do they affect the low scoring rate after AOEL.

### The relationship between the attack skills and the scoring rate

From the usage rate, the main skill for AIEL is the backhand flip and the usage rate is approximately 50%, which is far higher than that of other skills. Other skills with usage rates exceeding 10% are the forehand flip and forehand drive. The usage rate of backhand drives and pivot forehand drives is fairly low. Regarding AOEL, the usage rate of backhand drives is the highest while the usage rate of pivot forehand drives is the lowest.

Combined with the scoring rate, the scoring rate of players of CTTT is quite high at over 90%, but the usage rate is quite low when the forehand drive, backhand drive and pivot forehand drive to AIEL are used. In addition, the scoring rate is approximately 60% when the backhand flip to AIEL is used, and its usage rate is very high. However, in the case of AOEL, the scoring rate is very low at less than 15%, regardless of what skills they use.

### The relationship between the attack timing and the scoring rate

According to the figures above, the scoring rate of AIEL in the matches is related to the attack timing. The scoring rate of different attack timings include the third stroke and the fifth stroke in STAS, the receive and the fourth stroke in RTAS, and the sixth stroke and later in SS.

In other words, the scoring rate decreases from the STAS to RTAS and then to SS. Moreover, within the same section, the earlier the attack timing is, the higher the scoring rate will be.

## Discussion

### The principle of obvious difference in scoring rate between AIEL and AOEL

Fast speed is one of the outstanding characteristics of table tennis [15]. Previous studies have shown that the maximum hitting speed of male table tennis players on the Beijing table tennis team can reach 18.40 m/s and the speed of the ball they hit can reach 12.64 m/s after it falls on the other side's playing surface [16]. The table-tennis table is only 2.74 m long and according to this speed, it takes only 0.15–0.22 s for the ball to go from one end of the table to the other. This is solely based on the evaluation of provincial team-level athletes. In addition, higher-level athletes may have faster ball speeds and need a shorter time to hit the ball and have it pass the table. The average human reaction time is greater than 0.3 s [17]. That is, the speed of the ball from one end of the table to the other end may exceed the reaction ability of an average person.

We often see that in high-level table tennis competitions, the players on both sides have a wonderful standoff and the ball speed is very fast. This is because high-level table tennis players have formed a stable skill system through long-term and systematic training [18, 19]. The key factors supporting their fierce confrontation in high-level competitions may include accurate prediction ability and stable conditioned reflex [20, 21]. With accurate prediction ability, a player can judge the line or landing point of the opponents' ball in advance and then make corresponding skill actions through a stable conditioned reflex to return the ball with high quality. For international table tennis players, the stable conditioned reflex is present and the main problem may be accurate prediction. An additional outstanding characteristics of table tennis is change [22]. In high-level table tennis competition, there can be various forms of change such as a variety of landing point changes, rotation changes, speed changes, strength changes and so on. This makes it difficult for players to predict accurately. With more changes, it becomes more difficult for players to predict, resulting in lower accuracy. In contrast, the players have less difficulty predicting and thus higher accuracy when fewer changes are present, which explains the results of this study.

As a result, the scoring rate is very high after AIEL, but low after AOEL. However, there are relatively fewer ways of dealing with the balls outside of the end line, at which point players can only make limited changes in the routes. Even when dealing with some high-quality balls outside of the end line, it is difficult to make simple route changes [9]. Therefore, when AIEL is performed, the opponents' prediction is relatively difficult, which may affect the quality of return stroke to a great extent. At the same time, the opponents' return is already dealing with the topspin ball out of the end line, and the means of return is relatively uniform. The difficulty of prediction is relatively low when the ball is again returned, resulting in a relatively high scoring rate. Similarly, the skills and tactics are relatively single when AOEL is performed; the opponents' prediction difficulty is relatively low, so they may return the ball with high quality, which may reduce the players' scoring rate. The results of this study show that whether the opponent is HO or LO, the scoring rate after AIEL and AOEL shows a similar trend. The scoring rate is relatively high after AIEL but low after AOEL. To an extent, it verifies that the phenomenon discussed in this study is common in high-level table tennis competitions.

### Tactic arrangement strategy in high-level table tennis competition

#### Take advantage of AIEL and force the opponents to AOEL

**Fig. 3:**
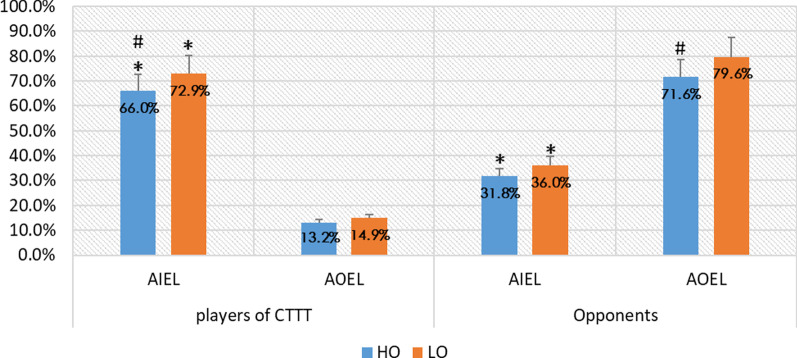
The scoring rate after attacking at different positions. *p < 0.5 (between different attack positions); ^#^*p* < 0.5 (between different groups)

According to the results of this study, the scoring rate after AIEL is significantly higher than the scoring rate after AOEL (Fig. 3). This phenomenon has little relationship with the types of skills used during attack and the key point is the position of attack (Table 1). Therefore, we must pay attention to this rule in tactical training and arrangement.

**Table 1:** Statistical table of the relationship between attack skills and scoring rate

Scoring rate of players of CTTT	HO		LO	
	Scoring rate (%)	Usage rate (%)	Scoring rate (%)	Usage rate (%)
AIEL				
Backhand flip	58.9^abcd#^	48.4	66.2^abcd^	56.2
Forehand flip	51.4^bcd^	10.2	56.6^bcd^	8.5
Forehand drive	95.9	10.4	96.1	12.1
Backhand drive	100.0	2.7	100.0	6.5
Pivot forehand drive	100.0	2.6	94.1	2.7
AOEL				
Forehand drive	12.9^f^	9.9	13.8	4.6
Backhand drive	11.1^f^	12.8	10.9	7.3
Pivot forehand drive	22.7^**#**^	3.1	33.3	1.9

^a^*p* < 0.5 (compared with forehand flip)

^b^*p* < 0.5 (compared with forehand drive)

^c^*p* < 0.5 (compared with backhand drive)

^d^*p* < 0.5 (compared with pivot forehand drive)

^e^*p* < 0.5 (compared with backhand drive)

^f^*p *< 0.5 (compared with pivot forehand drive)

^#^*p* < 0.5 (between different groups).

**Fig. 4:**
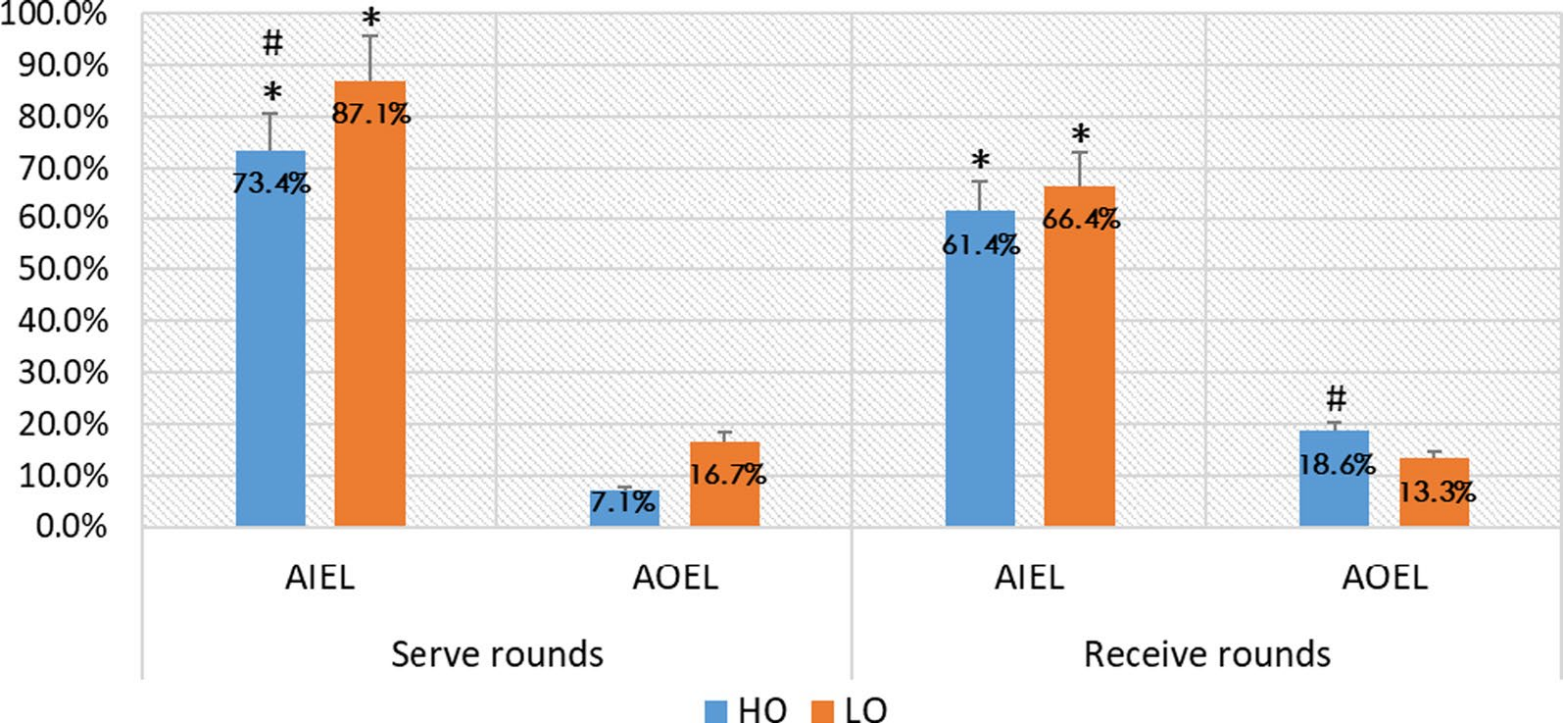
The relationship between the rounds and scoring rate after attacking at different positions. *p < 0.5 (between different attack positions); ^#^*p* < 0.5 (between different groups)

Moreover, the scoring rate is more than 70% in the serve rounds and just over 60% in the receive rounds, which indicates a certain gap (Fig. 4). Perhaps the reason is that players have the right to control the serve in the serve round, and there are many changes in the rotation and landing point of the serve. Through high-quality service, opponents' AIEL can be limited. It can be seen from the data that the scoring rate after attacking the short ball served by the opponents is significantly lower than that after attacking the short ball pushed by the opponents (Table 1), which supports the statement above.

In addition, the scoring rate of AOEL was not high (Fig. 3). Therefore, players should try their best to avoid AOEL or let the opponents AOEL. However, they should not blindly give the opponents the ball out of the end line or blindly let them AOEL. In Part 4.1 of this study, the reasons for the high scoring rate of AIEL were discussed. There can be more changes when AIEL, which can make opponents' prediction more difficult. When a player blindly gives the opponents the ball out of end line, the difficulty of prediction will be low and the opponent may be able to easily return every ball. Therefore, when we use the control skills to return the ball, we should not blindly give the opponents the ball out of the end line and we need to combine the ball in and out of the end line.

#### Pay attention to the timing of attack

**Fig. 5:**
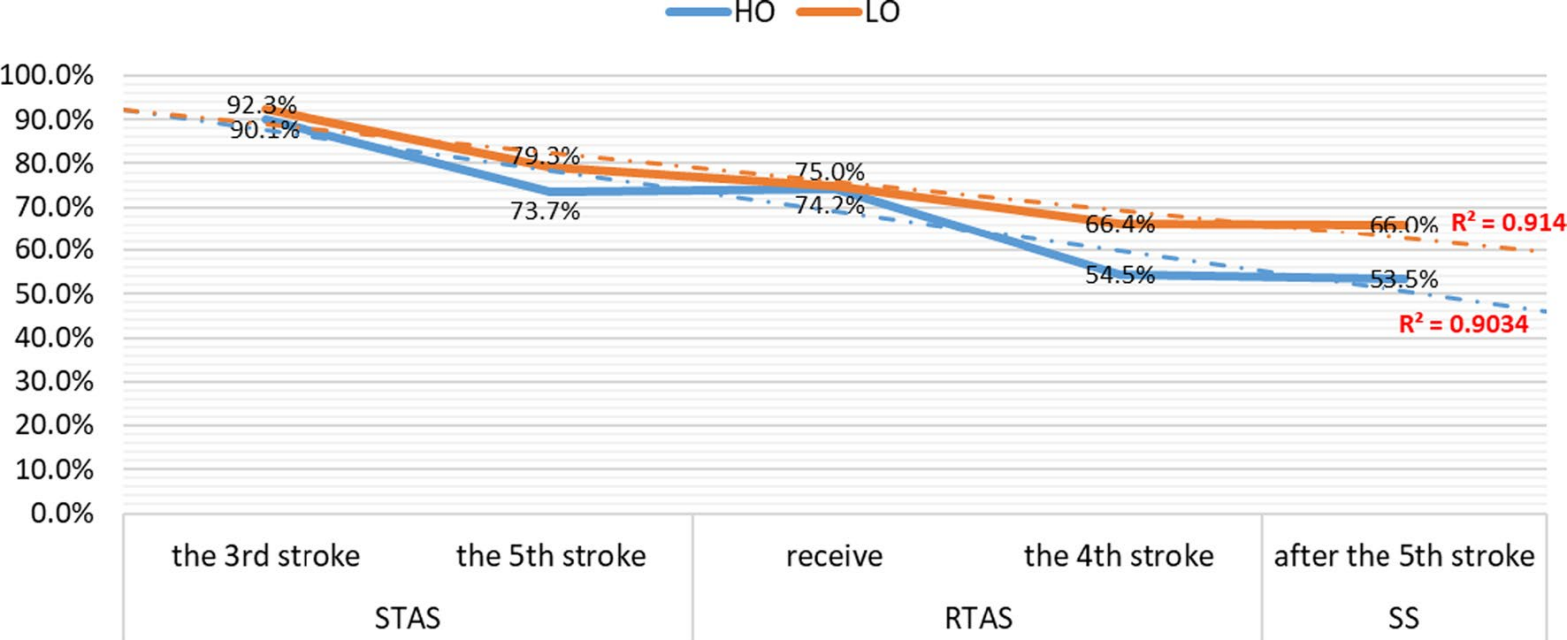
The relationship between attack timing and his scoring rate after AIEL

In high-level table tennis competition, the players should pay attention to the timing of attack and seize the opportunity to AIEL as soon as possible. The results of this study show that there is a relationship between the scoring rate after AIEL and the timing of attack, whether they are playing against the HO or LO. As seen in the different sections, the scoring rate after AIEL in STAS is the highest, and that in RTAS is the second highest. Furthermore, in any section, an earlier attack time results in a higher scoring rate (Fig. 5). Generally, most points in a table tennis match start with the short ball (the ball lands near the net or the half-court, with the second landing point still on the table); one of the players attacks with it and then the two players enter into topspin confrontation until one player wins. Therefore, with the increasing strokes of short balls that the two players control at one point, the psychological preparation for the opponent's AIEL is also increasingly sufficient and the effect of returning to the opponent's AIEL is naturally improved.

#### Keep an eye on every half-court shot and deep service from the opponents

After the opponents' short service or drop shot, the scoring rate after AIEL was approximately 60%. However, when the opponents serve or push the half-court shot, the scoring rate after AIEL is greater than 97% (Table 1). Based on the data above, a half-court shot has a very good chance to score. The half-court shot is relatively long and the second landing point is near the end line, therefore the speed of the ball is not as fast as deep shot; this allows more time to judge than when the ball is out of the end line. Moreover, for the half-court shot, players can use the skills of forehand drive, backhand drive and pivot forehand drive. Compared with the backhand flip and forehand flip to AIEL, the hitting force and quality are significantly greater, the threat to the opponent is bound to be greater, and the scoring rate is bound to be higher.

#### Integrate the rules of attack into daily training

According to the above discussion, it is apparent that when formulating the tactical strategies for table tennis matches; players can take advantage of AIEL and force opponents to AOEL, pay attention to the timing of attacks, and keep an eye on every half-court shot and deep service from opponents. These rules of attack look simple but are difficult to execute. Players must integrate the rules of attack into training and carry out special training in their daily training. Coaches should integrate the rules of attack into the guiding ideas of training and make athletes form the tactical awareness of attack through corresponding multi-ball training. At the same time, physical trainers should cooperate to train for strength and reaction ability to improve the quality and stability of athletes' attacks.

## Conclusions and suggestions

### The conclusions

In high-level table tennis competitions, the rules of attack in table tennis tactics discovered by the coaches and researchers of CTTT are scientific. According to the rules, the complicated skills and tactics can be simplified into two concepts: AIEL and AOEL. That is, players only need to take advantage of AIEL and force opponents to AOEL in matches. In terms of the details, players need to pay attention to the timing of AIEL. The earlier players attack, the better effect it will have. At the same time, players should make full use of the good effects of deep shots in the RTAS and seize the opportunities for half-court shots to obtain scores.

### The suggestions

The training of skills and tactics for high-level table tennis players can be carried out based on the rules of attack. Coaches and players need to have a deep understanding of the core points of AIEL and AOEL. At the same time, coaches and players should pay attention to the ability of AIEL in the training, especially the ability to attack the half-court shot and integrate these into players' awareness of skills and tactics.

## Data Availability

The datasets used and/or analysed during the current study are available from the corresponding author on reasonable request.

## Abbreviations

CTTT: Chinese table tennis team
AIEL: Attack in the end line
AOEL: Attack out of the end line
HO: High-level opponents (ranked top 30 in the world by the International Table Tennis Federation)
LO: Low-level opponents (ranked below 30 in the world by the International Table Tennis Federation)
STAS: The serve-then-attack section (including the service, the third stroke, the fifth stroke)
RTAS: The receive-then-attack section (including the receive and the fourth stroke)
SS: The stalemate section (the sixth stroke and later)
